# The Use of Alcohol and Knowledge of Cardiovascular Diseases among Ellisras Rural Children Aged 14–22 Years: Ellisras Longitudinal Study

**DOI:** 10.3390/ijerph16152650

**Published:** 2019-07-24

**Authors:** Moloko Matshipi, Kotsedi D. Monyeki, Norman Mafumo, Suzan M. Monyeki, Hlengani J. Siweya, Han C. G. Kemper

**Affiliations:** 1 Department of Human Physiology and Environmental Health, University of Limpopo, Sovenga 0727, South Africa; 2 Department of Curriculum Studies, School of Education, University of Limpopo, Sovenga 0727, South Africa; 3 Executive Dean, Faculty of Science and Agriculture, University of Limpopo, Sovenga 0727, South Africa; 4 Amsterdam UMC, Amsterdam Health Research Institute, 1081 HV Amsterdam, The Netherlands

**Keywords:** Ellisras Longitudinal Study, cardiovascular diseases, children, alcohol use

## Abstract

The harm alcohol abuse does to physical and mental health is well established. The perception of cardiovascular disease risk factors and alcohol use requires attention. This study aims to investigate the association between alcohol usage and knowledge of cardiovascular diseases (CVDs) risk factors among Ellisras rural adolescents and young adults aged 14–22 years. In this cross-sectional study a total of 1409 subjects (736 boys and 673 girls), aged 14–22 years, from the Ellisras Longitudinal Study, South Africa completed a validated alcohol use and CVDs knowledge questionnaire. Logistic regression was used to estimate the association. The prevalence of alcohol intake increased with increasing age among girls (13.6% to 17.7%) and boys (10.3% to 16.9%) and reached a statistically significant difference (*p* < 0.024) at an older age category (20–22 years). There was a significant (*p* < 0.05) association between alcohol use and a positive response on the following knowledge statements: The fact that cardiovascular disease attacked all age groups and mostly elderly people (the odds ratio (OR) ranged between 0.5 95% confidence interval (CI) = 0.33–0.74 and OR = 2.86 95% CI = 1.27–6.42). Medical doctors can help to diagnose somebody with cardiovascular diseases (OR ranged between 2.25 95% CI = 1.49–3.39 and OR = 0.75 95% CI = 0.65–0.87). The condition for cardiovascular diseases developed over a long period (OR ranged between 1.75 95% CI = 1.16–2.64 and OR = 2.23 95% CI = 1.34–4.07). The prevalence of alcohol use in Ellisras rural adolescents and young adults begins between the ages of 14 and 16 years and increases with age. Binge drinking was more evident on Fridays and Saturdays among the Ellisras rural adolescents and young adults with girls showing a significantly higher prevalence of binge drinking compared to boys on a Friday.

## 1. Introduction

Alcohol is a commonly consumed drug in the rural South African population. It is perceived to have a protective lipids effect, although the sources of such information are baseless [1]. However, excessive alcohol consumption has negative effects on the body such as arterial fibrillation, high blood pressure, elevated glucose and lipids level, and obesity [2,3]. Furthermore, researchers suggest that alcohol consumption may represent a sizeable risk factor for excessive weight gain [3,4], though knowledge of the calorie content of alcohol and its food equivalence is still lacking, particularly with homemade beer, which is consumed quite often by the rural South African population. It has been reported previously that South Africa has the highest levels of alcohol consumption per adult drinker than any other region in the world [5]; and that risky drinking patterns start at the age of 15–24 and continue into adulthood [6], thus making this group a target for intervention.

Cardiovascular diseases (CVDs) risk factors are on the increase because of the adoption of a western lifestyle in Africa [7]. Previous studies have reported limited knowledge of some of the CVDs risk factors such as warning signs and symptoms (slurred speech, dizziness, numbness, weakness, headache, vision problem) among the Nigerian population due to a low level of education [7,8,9,10,11]. Reducing the interval between the awareness of warning signs and symptom recognition of CVDs risk factors and the decision to seek medical care could assist in preventing these CVD deaths [12], which are labelled as witchcraft deaths in the rural South African population [13].

In counselling patients regarding alcohol intake, it is important to understand the public use of alcohol, knowledge of CVD risk factors and the risks those perceptions pose to the actual alcohol drinking behaviour. The purpose of the study was therefore to investigate (i) the prevalence of alcohol usage, (ii) knowledge of CVDs risk factors, and finally, (iii) the association between alcohol usage and knowledge of CVDs risk factors among the Ellisras rural population aged 14–22 years.

## 2. Materials and Methods

### 2.1. Study Design and Sampling

The current study was a cross-sectional study that is part of an ongoing Ellisras Longitudinal Study (ELS). The ELS design and sampling methodology was reported elsewhere [14]. A total of 1409 adolescents and young adults (736 boys and 673 girls) aged between 14 and 22 years who are part of the ELS participated in the study during the period from 18 September 2008 to 11 February 2009 in addition to the 1654 (854 boys and 800 girls) who participated in the smoking study [15] of 1 March–3 May 2005. The Ethics Committee of the South African Medical Research Council granted ethical approval prior to the survey and the parents or guardians provided informed consent. Parents of younger subjects (≤17 years) signed the assent form while older subjects (≥18 years) signed the consent form after receiving a verbal explanation of the study contents from the project’s principal investigator.

### 2.2. Data Collection

A validated survey instrument to assess the frequency and quantity of alcohol intake was used to collect data of alcohol intake in a rural South African population [16,17]. The CAGE questions developed by Ewing [18] were used in the study. The CAGE acronym stands for four yes/no items constituting the screening test: (1) Have you ever felt that you ought to **C**ut down on your drinking? (2) Have people **A**nnoyed you by criticising your drinking? (3) Have you ever felt bad or **G**uilty about your drinking? (4) Have you ever had a drink first thing in the morning to steady your nerves or to get rid of a hangover (**E**ye-opener). The total score ranged from 0 to 4. The recommended cutoff for CAGE used in the current study is ≤2 to screen for alcohol abuse or dependence [19]. The prevalence of alcohol use, amount of alcohol taken, and number of days on which people were seen drinking near schools are recorded in Table 1, Table 2 and Table 3, respectively. 

### 2.3. Knowledge of CVDs Risk Factors

Knowledge of CVDs risk factors [8,9,10,11] among the Ellisras rural adolescent and young adults included questions such as those listed in Table 4. In the statement provided, the subjects had the options of yes, no, or not sure.

The principal investigator translated the alcohol and CVDs questionnaire from English into the two local spoken languages (Northern Sotho and Setswana) with the help of Ellisras local teachers and, then, with help from other new Northern Sotho and Setswana speaking teachers in the Ellisras area, the questionnaires were then translated back into English. The translation back to English showed no disparity with the Northern Sotho and Setswana languages (Ellisras rural area local languages). In a pilot study conducted prior to the survey, face, content, and construct validity were adequately attended to [16,17,20].

### 2.4. Definition of Variables

Participants’ drinking behaviour was assessed in the questionnaire according to the following questions: Have you ever drunk alcohol? (By alcohol we mean sorghum beer, western style beer, wine, spirit, brandy, cider, etc.) (yes/no = 1/0); Have you experimented, but are not currently drinking? (yes/no = 1/0); Do you drink alcohol now? (once a year, twice a year, or three or four times a year = 0; once a month, twice a month, three or four times a month, once a week, twice a week, three or four times a week, or daily = 1). Have you ever drunk was identified if they answered that they tried once a year, twice a year, or three or four times a year coded as 0. Participants were considered as current drinkers if they answered that they drink once a month, twice a month, three or four times a month, once a week, twice a week, three or four times a week, or daily and were coded as 1.

Risky drinking was defined as drinking (Black Label, Castle, and Hansa) five or more bottles of 750 mL (has less than 5.5% alcohol/volume) per day for boys and three or more bottles of 750 mL (has less than 5.5% alcohol/volume) per day for girls [16,17]. While communal drinking, which was regarded as hazardous/harmful drinking, was measured in 1 L per serving [21]. Communal drinking occurs when two or more people share a drink from one source. Wine (red and white) 9%–17% alcohol), desert wine (14%–25%), vodka (35%–60% alcohol), brandy (35%–60% alcohol), gin (38%–50%), whiskey (40%, 43%, or 46%), and scotch (63.5%) were excluded from the analysis as the study participants indicated their intake of them was sporadic. 

### 2.5. Data Analysis

All statistical analyses were performed using the SPSS Version 14.0 (SPSS Inc., Chicago, IL, USA). The Shapiro–Wilk W test was used to test the normal distribution of the continuous variables and later descriptive statistics were run by gender. A chi-square test was used to compare two or more sets of nominal data that had been arranged into categories by frequency counts of large samples, while the Fisher’s exact test was used when the expected cell frequencies were small (less than five) [22]. Logistic regression analysis was applied to determine the association between current alcohol usage and knowledge of CVDs. In assessing the relationship between knowledge of CVDs and alcohol usage, the number of drinks consumed per week was used as the primary outcome. Covariates associated with the outcome were included in the model (age, gender, seeing community members drinking alcohol next to the school during school hours in the past 30 days (less than nine times = 0 and more than 10 times = 1)). The statistical significance was set at *p* < 0.05.

### 2.6. Ethics Approval and Consent to Participate 

The Ethics Committee of the South African Medical Research Council granted ethical approval prior to the study and the parents or guardians and subjects provided written informed consent prior to the study.

## 3. Results

Table 1 presents the descriptive statistics for the prevalence of alcohol use among Ellisras rural adolescence and young adults aged 14–22 years. The current prevalence of alcohol usage was high (8.2%–10.1%) among girls compared with boys (6.7%–6.8%) at adolescence and young adult age groups, though insignificant. Current alcohol drinkers exhibit insignificant risky drinking (from 2.0% to 5.6%) during the week to (5.5%–9.5%) over the weekend between boys and girls.

Table 2 shows the amount of alcohol taken by Ellisras rural adolescents and young adults aged 14–22 years during the week and on weekends. A number of Ellisras rural adolescents and young adults (over 5%) reported to be heavy drinkers during the week with very few subjects (0.3%) drinking on Sundays. Communal drinkers were high on weekends, Friday (4.9% for boys and 7.3% for girls) and Saturday (6.1% for boys and 7.7% for girls) with girls reaching a high significant level (*p* = 0.049) compared to boys on Friday.

Table 3 shows frequency and percentage frequency for the number of days ELS subjects saw community members drinking alcohol next to the school during school hours in the past 30 days. Almost all the subjects had community members drinking alcohol next to the school premises during school hours in the past 30 days. At least 96.7% of the girls had people drinking alcohol next to the school premises during school hours once or twice.

A positive response of having knowledge of cardiovascular diseases among Ellisras rural adolescence and young adults is presented in Table 4. There was no significant difference between boys and girls regarding the positive response provided to all CVDs questionnaires regarding knowledge. The majority (70.9% of boys and 69.5% for girls) of Ellisras rural adolescents and young adults were aware that smoking, lack of physical activity, fatty foods, and too much salt and sugar in the food could lead to cardiovascular disease. A low number of adolescents and young adults (31.8% for boys and 29.7% for girls) were aware that CVD does not have signs and symptoms they are only designated by a specific event like a heart attack. Knowledge that cardiovascular disease attacks in all age groups but mostly elderly people was positively answered by a few of the children (40.8% boys and 43.4% of girls).

Table 5 presents frequency and percentage frequency of the total score concerning knowledge of the CVD risk factors among the Ellisras adolescents and young adults. The total score obtained by children on the knowledge of CVD risk factors ranged from 0% to 22.5% of ELS adolescents and young adults who obtained slightly above-average scores. The majority of the subjects obtained less-than-average scores on the questionnaires.

Table 6 presents the odds ratio and 95% confidence interval for the association of alcohol usage, and knowledge of CVD risk factors among Ellisras rural adolescents and young adults unadjusted and adjusted for age and gender. There was a significant (*p* ranges between 0.000 and 0.008), inverse association between alcohol usage and positive response on the following knowledge statements even after adjusting for the covariate: The fact that cardiovascular disease attacked all age groups and mostly elderly people (OR ranged between 0.5 95% CI = 0.33–0.74 and OR = 2.86 95% CI = 1.27–6.42); Medical doctors can help to diagnose somebody with cardiovascular diseases (OR ranged between 2.25 95% CI = 1.49–3.39 and OR = 0.75 95% CI = 0.65–0.87). The condition for cardiovascular diseases developed over a long period (OR ranged between 1.75 95% CI = 1.16–2.64 and OR = 2.23 95% CI = 1.34–4.07). However, the statement “there are no component causes for cardiovascular diseases as they are associated with lifestyle” was associated with current alcohol drinking after adjusting for covariates (OR = 0.31, 95% CI = 0.1–1.02).

## 4. Discussion

Reliable and valid information on the prevalence of alcohol usage among Ellisras rural adolescents and young adults is an important contribution to public health policy. The results of this investigation indicate that the prevalence of alcohol usage increases with age, with girls reaching a highly significant difference as compared to boys in the 20–22-years-old group, boys reached a lowest prevalence of consumption in the 20–22-years-old group. Drinking alcohol for teenage girls was associated with high teenage pregnancy, which has economic benefits among rural South African youth [21,23]. Furthermore, South African National data suggest that a culture of sporadic heavy or binge drinking among young people may be spreading to rural areas [16,17,21], which is confirmed by the current study.

A previous study reported that risky alcohol consumption starts at age 15 and increases with increasing age [6]; this study partially corroborates the previous finding; however, an interesting trend is observed in boys, where there is a reduction in alcohol usage from age 20 to 22. Previous studies have reported that education [24], employment [25], and financial capacity [26] determine the pattern and frequency of alcohol use. The difference in education level and financial capacity among the studied age groups may account for this observation, although it remains to be investigated whether it applies in this population.

The fact that girls in the current study drink more than boys raises a serious concern. Plausible explanations for these trends lie in the areas of access, poor community policing, large-scale youth specific marketing, advertisement, poverty, and lack of recreational facilities in the rural communities. Furthermore, the risk for youth drinking and binge drinking is mainly caused by environmental stressors such as poverty, unemployment, and crime [21]. Girls become the head of the family at a younger age given the breaking down of families due to HIV and AIDS in present day rural South Africa, hence the exposure to alcohol for economic reasons [16,23,27].

The majority of adolescents showed an onset age of 14–16 years for alcohol abuse. Researchers in the US have found that the earlier the age at which people begin drinking, the more likely they are to become alcohol dependent later in life [28,29]. Those that begin drinking in their teen years are also more likely to experience alcohol-related unintentional injuries than those who begin to drink at a later age [30,31]. It is possible that people who engage in a variety of deviant or illegal alcohol drinking behaviours at an early age are more likely to continue later in life [32]. Prospective research found a younger age of initiation to be strongly associated with a high level of alcohol misuse at age 17 years and adverse health and social profiles later in life [1,20,33].

A majority of the participants in the current study show average and below-average scores in the knowledge of CVDs risk factors questionnaire, which clearly shows that the level of CVDs risk factors knowledge was low in this population. Similar results were reported [7,34,35] among Nigerian and Brazilian populations, while Whitman et al. [1] reported an above-average score in the knowledge of CVDs among USA participants. In the current study, we found a positive association between alcohol use and some knowledge of CVDs risk factors. Although alcohol use may be perceived to have a protective lipid effect, this is mostly information from lay media [1]; alcohol can, actually, increase the risk of hypertension and arterial fibrillation and increases glucose levels in the blood [1,36,37,38,39]. Consequently, the lack of controlled trials on the actual CVDs benefits of alcohol and the conflicting reports on health effects of moderate consumption of alcohol speculation will continue in the topic [1,7] particularly, in the rural South African population wherein CVDs knowledge level was low with increasing alcohol use as it was the case in the current study.

Our study has some limitations that could affect the generalisability of our study. The data were not representative of the adolescent and young adult population of Ellisras rural population. In particular, some ELS subjects could have decided not to be available for the study as they were currently involved in drinking alcohol of which their parents or older siblings in the family were not aware. Second, the amount of alcohol consumed was derived from self-reporting questionnaires, which could introduce recall bias in the conclusion of the results. We did not separate the types of alcohol consumed, as individuals could drink four to five different types of alcohol (that is, beers such as Castle, Castle light, Hansa, Black Label, Milk Stout, and homemade beer) in one sitting as a result of communal drinking. It is worthwhile noting that communal drinking patterns are directed by the person who has the money to sponsor the group at that moment and changes from one person to the other in one sitting in rural Ellisras area. Finally, the current study was cross-sectional in nature and therefore cause and effect relationships could not be established.

## 5. Conclusions

The prevalence of alcohol in Ellisras rural adolescents and young adults starts between 14 and 16 years and increases with increasing age in girls. Young adult girls showed a significantly higher prevalence of binge drinking compared to boys on a Friday. There was a significant association between positive answers to most of the questions on knowledge of CVDs risk factors and alcohol use in the current study. Although many showed above-average knowledge of CVDs risk factors, more effort directed at educating the Ellisras rural adolescence and young adults about the dangers of CVDs risk factors can still be beneficial as some of the important questions on CVDs risk factors still showed below-average knowledge.

## Figures and Tables

**Table 1:** Prevalence of alcohol usage and CAGE among Ellisras rural adolescence and young adults aged 14–22 years.

	14–16 Years			17–19 Years			20–22 Years		
	Boys	Girls		Boys	Girls		Boys	Girls	
Population Characteristic									
*N*	101	89		325	302		310	282	
	% (n)	% (n)	*p*-value	% (n)	% (n)	*p*-value	% (n)	% (n)	*p*-value
Age in years (mean ± SD) *	16.0 (0.82)	16.0 (0.70)	0.955	18.8 (0.84)	18.8 (0.83)	0.857	21.0 (0.65)	21.0 (0.66)	0.789
Alcohol Use Questions									
Have you ever drunk alcohol?	14.9 (15)	14.6 (13)	0.967	16.9 (55)	13.6 (41)	0.319	10.3 (32)	17.7 (50)	0.024
Are you currently drinking alcohol?	6.9 (7)	10.1 (9)	0.604	10.8 (35)	7.9 (24)	0.271	6.8 (21)	9.2 (26)	0.310
Do you smoke a lot when you drink?	2.0 (2)	5.6 (5)	0.201	6.5 (21)	3.6 (11)	0.128	2.6 (8)	5.0 (14)	0.140
CAGE Questions									
(1) Have you ever felt that you ought to **C**ut down on your drinking?	12.9 (13)	13.5 (12)	0.913	16 (52)	12.9 (39)	0.343	8.4 (26)	15.6 (44)	0.016
(2) Have people **A**nnoyed you by criticising your drinking?	3.0 (3)	6.7 (6)	0.245	8.6 (28)	0.7 (2)	0.000	3.2 (10)	3.9 (11)	0.669
(3) Have you ever felt bad or **G**uilty about your drinking?	6.9 (7)	10.1 (9)	0.469	11.4 (37)	9.6 (29)	0.513	7.7 (24)	11.7 (33)	0.139
(4) Have you ever had a drink first thing in the morning to steady your nerves or to get rid of a hangover (**E**ye-opener)?	5.9 (6)	10.1 (9)	0.326	9.2 (30)	8.6 (26)	0.803	7.1 (22)	11.7 (33)	0.079
Prevalence of CAGE&0.803									
Alcohol abuse	42.9 (3)	88.9 (8)	0.095	94.3 (33)	79.2 (19)	0.107	76.2 (16)	76.9 (20)	0.356
Current Alcohol Drink									
Risky behaviour on weekdays	2.0 (2)	5.6 (5)	0.201	5.5 (18)	4.0 (12)	0.382	3.2 (10)	2.8 (8)	0.789
Risky behaviour on weekends	6.9 (7)	10.1 (9)	0.469	9.5 (31)	7.0 (21)	0.280	5.5 (17)	7.1 (20)	0.448

* Mean and standard deviation, *t*-test used, Fisher exact tests, & = above the cut off point for the CAGE questionnaire based on Dhalla and Kopec, [17].

**Table 2:** The amount of alcohol taken by Ellisras rural children (boys, *n* = 102 and girls, *n* = 104) aged 14–22 years during the week and on weekends.

Amount of Alcohol		Week			Friday			Saturday			Sunday		
Boys	Girls	Boys	Girls		Boys	Girls		Boys	Girls		Boys	Girls	
		% (n)	% (n)	*p*-value	% (n)	% (n)	*p*-value	% (n)	% (n)	*p*-value	% (n)	% (n)	*p*-value
1–2 drinks/day	<1 drink/day	1.9 (14)	2.5 (17)	0.651	3.3 (24)	2.5 (17)	0.426	2.0 (15)	2.1 (14)	0.956	1.4 (10)	1.0 (7)	0.575
3–4 drinks/day	2–3 drinks/day	3.5 (26)	3.9 (26)	0.950	2.7 (20)	3.1 (21)	0.602	2.6 (19)	2.8 (19)	0.785	0.8 (6)	0.7 (5)	0.561
5 or more drinks/day	4 or more drinks/day	5.3 (39)	5.6 (38)	0.856	3.0 (22)	2.5 (17)	0.607	3.1 (23)	2.8 (19)	0.747	0.3 (2)	0.3 (2)	0.654
Communal drinks ^&^	Communal drinks ^&^	3.1 (23)	3.4 (23)	0.953	4.9 (36)	7.3 (49)	0.049	6.1 (45)	7.7 (52)	0.265	11.4 (84)	13.4 (90)	0.810

One drink was measured as 750 mL (5.5% alcohol), ^&^ traditional and western beer, passing around a litre-size container and several people drink from the same container.

**Table 3:** Frequency and percentage frequency for the number of days Ellisras Longitudinal Study (ELS) subjects saw community members drinking alcohol next to the school during school hours in the past 30 days.

	14–16 Years		17–19 Years		20–22 Years	
	Boys	Girls	Boys	Girls	Boys	Girls
Number of days	101	89	325	302	310	282
	% (n)	% (n)	% (n)	% (n)	% (n)	% (n)
1–2	56.4 (57)	66.3 (59)	81.8 (266)	96.7 (292)	77.4 (240)	97.5 (275)
3–5	14.9 (15)	2.2 (2)	6.2 (20)	0.7 (2)	10.3 (10.3)	1.4 (4)
6–9	8.9 (9)	7.9 (7)	3.1 (10)	0.7 (2)	4.8 (15)	0.7 (1)
10–19	13.9 (14)	15.7 (14)	6.8 (22)	1 (3)	5.2 (16)	0.4 (1)
20–30	5.9 (6)	7.9 (7)	2.2 (7)	1 (3)	2.3 (7)	0.4 (1)

**Table 4:** Percentage frequencies, frequencies, and *p*-values for positive response on knowledge of cardiovascular diseases (CVDs) among Ellisras rural children aged 14–22 years.

CVDs Knowledge Statements	Boys *n* = 736	Girls *n* = 636	
	% (n)	% (n)	*p*-values
Cardiovascular disease attacks all age groups and mostly elderly people	40.8 (300)	43.4 (292)	0.524
The conditions linked to cardiovascular disease are multi-factorial—genetics and lifestyle	69.0 (508)	68.5 (461)	0.928
Smoking, lack of physical in activity, fatty food, and too much salt and sugar in the food could lead to cardiovascular diseases	70.9 (522)	69.5 (468)	0.812
Cardiovascular diseases are associated with stress, overweight, obesity, high blood pressure, lack of physical activity, and too much smoking	58.7 (432)	58.2 (392)	0.930
Medical doctors can help to diagnose somebody with cardiovascular diseases	55.4 (408)	54.8 (369)	0.902
There are no specific signs and symptoms of cardiovascular diseases as they are only designated by specific events e.g., heart failure	31.8 (234)	29.7 (200)	0.540
The conditions for cardiovascular disease develop over a long period	62.0 (456)	59.4 (400)	0.632
Usually people with high blood pressure and diabetes experience cardiovascular diseases	68.6 (505)	65.2 (439)	0.548
Cardiovascular diseases have high public importance and people take preventative measures seriously in our community such as giving up smoking, exercising often, and eating a lot of vegetables and fruits.	62.6 (461)	64.0 (431)	0.796
There are no component causes for cardiovascular diseases as they are diseases associated with lifestyle	65.6 (483)	64.3 (433)	0.816

**Table 5:** The frequency and percentage frequency of the total score on perception of cardiovascular risk factors among Ellisras adolescents and young adults.

	14–16 Years			17–19 Years			20–22 Years		
	Boys	Girls		Boys	Girls		Boys	Girls	
Score	101	89		325	302		310	282	
	% (n)	% (n)	*p*-value	% (n)	% (n)	*p*-value	% (n)	% (n)	*p*-value
0	2.0 (2)	3.4 (3)	0.561	3.1 (10)	4.6 (14)	0.328	4.2 (13)	2.5 (7)	0.266
1	4.0 (4)	3.4 (3)	0.836	7.1 (23)	6.6 (20)	0.834	5.2 (16)	3.5 (10)	0.359
2	14.9 (15)	5.6 (5)	0.062	8.0 (26)	8.6 (26)	0.779	11.6 (36)	13.1 (37)	0.623
3	13.9 (14)	21.3 (19)	0.255	16.9 (55)	19.2 (58)	0.536	17.4 (54)	16.3 (46)	0.762
4	13.9 (14)	15.7 (14)	0.755	12.6 (41)	13.6 (41)	0.755	13.5 (42)	11.3 (32)	0.476
5	20.8 (21)	19.1 (17)	0.812	17.8 (58)	19.2 (58)	0.717	18.7 (58)	20.2 (57)	0.705
6	21.8 (22)	22.5 (20)	0.927	20.0 (65)	18.5 (56)	0.704	17.4 (54)	20.6 (58)	0.420
7	6.9 (7)	5.6 (5)	0.727	8.6 (28)	5.3 (16)	0.130	7.7 (24)	9.2 (26)	0.553
8	2.0 (2)	1.1 (1)	0.642	4.0 (13)	2.3 (7)	0.246	1.9 (6)	1.8 (5)	0.836
9	0 (0)	1.1 (1)	0.288	1.2 (4)	1.7 (5)	0.660	1.0 (3)	1.1 (3)	0.908
10	0 (0)	1.1 (1)	0.288	0.6 (2)	0.3 (1)	0.608	1.3 (4)	0.4 (1)	0.278

**Table 6:** Odds ratio and 95% confidence interval for the association of alcohol usage and the knowledge of cardiovascular diseases among Ellisras rural adolescence and young adults (boys, *n* = 736 and girls, *n* = 673) aged 14–22 years.

CVDs Knowledge Statements	OR	95% CI		*p*-Value	OR *	95% CI *		*p*-Value *
(a) Cardiovascular disease attacks all age groups and mostly elderly people	0.50	0.33	0.74	0.001	2.86	1.27	6.42	0.011
(b) The conditions linked to cardiovascular disease are multi-factorial—genetics and lifestyle	1.05	0.70	1.57	0.822	0.23	0.03	1.53	0.128
(c) Smoking, lack of physical activity, fatty food, and too much salt and sugar in the food could lead to cardiovascular diseases	0.65	0.44	0.95	0.027	0.37	0.07	1.95	0.239
(d) Cardiovascular diseases are associated with stress, overweight, obesity, high blood pressure, lack of physical activity, and too much smoking	0.58	0.35	0.97	0.037	0.85	0.51	1.44	0.554
(e) Medical doctors can help to diagnose somebody with cardiovascular diseases	2.25	1.49	3.39	0.000	0.75	0.65	0.87	0.000
(f) No signs and symptoms of cardiovascular diseases as they are only designated by specific events e.g., heart failure	1.08	0.74	1.58	0.693	0.76	0.46	1.26	0.288
(g) The conditions for cardiovascular disease develop over a long period	1.75	1.16	2.64	0.008	2.33	1.34	4.07	0.003
(h) Usually people with high blood pressure and diabetes experience cardiovascular diseases	1.01	0.06	1.68	0.974	0.69	0.34	1.38	0.292
(i) Cardiovascular disease has a high public importance and people take preventative measures seriously in our community like giving up smoking, exercising frequently, and eating a lot of vegetables and fruits.	1.78	1.20	2.63	0.004	1.38	0.83	2.29	0.222
(j) There are no component causes for cardiovascular diseases as they are diseases associated with lifestyle	0.56	0.25	1.22	0.143	0.311	0.10	1.02	0.053

* Adjusted for covariate (age, gender, seeing community members drinking alcohol next to the school during school hours the past 30 days), total knowledge score of cardiovascular diseases were controlled for in logistic regression analysis.

## References

[1] Whitman I.R., Plectcher M.J., Vittinghof E., Imbugia K.E., Macuire C., Bettencourt L., Sinha T., Parsnick T., Tison G., Mulvanny C.G. (2015). Perception, information source, and behaviour regarding alcohol and heart health. Am. J. Cardiol..

[2] Larsson S.C., Drea N., Wolk A. (2014). Alcohol consumption and risk of atrial fibrillation: A prospective study and dose response meta-analysis. J. Am. Coll. Cardiol..

[3] Xin X., He J., Frontinin M.G., Ogden L.G., Motsamai O.I., Whelton P.K. (2001). Effect of alcohol reduction on blood pressure: A meta-analysis of randomise control trials. Hypertension.

[4] Hingson R.W., Heeren T., Jamanka A., Howland J. (2000). Age of drinking onset and unintentional injury involvement after drinking. JAMA.

[5] Peltzer K., Davids A., Njuho P. (2011). Alcohol use and problem drinking in South Africa: Findings from a national population-based survey. Afr. J. Psychiatry.

[6] Rehm J., Rehn N., Room R., Monteiro M., Gmel G., Jernigan D., Frick U. (2003). The global distribution of average volume of alcohol consumption and patterns of drinking. Eur. Addict. Res..

[7] Ramsoomar L., Morojele N.K. (2012). Trend in alcohol prevalence, age of initiation and association with alcohol related harm among South African youth: Implication for policy. SAMJ.

[8] Chikere E.I.C., Mayowa M.O. (2011). Prevalence and perceived health effect of alcohol use among male graduate students in Owerri, South-East Nigeria: A descriptive cross-sectional study. BMC Public Health.

[9] Reeves M.J., Rafferty A.P., Aranha A.A., Theisen V. (2008). Changes in knowledge of stroke risk factors and warning signs among Michigan adults. Cereb. Dis.

[10] Das K., Mondal G.P., Dutta A.K., Mukherjee B., Mukherjee B.B. (2007). Awareness of warnings symptoms and risk factors of stroke in the general population and in stroke survivors. J. Clin. Neurosci..

[11] Borges T.T., Rombaldi A.J., Knuth A.G., Hallal P.C. (2009). Knowledge on risk factors for chronic diseases: A population-based study. Cad. Saude Publ..

[12] Obembe A.O., Olaugun M.O., Bamikole A.A., Komolafe M.A., Odetunde M.O. (2014). Awareness of risk factors and warning signs of stroke in Nigerian University. J. Stroke Cereb. Dis..

[13] Monyeki K.D., Kemper H.C.G., Twisk J.W.R., O’Dea J.A., Eriksen M. (2010). Trends in obesity and hypertension in South African youth. Childhood Obesity Prevention.

[14] Monyeki K.D., Van Lenthe F.J., Steyn N.P. (1999). Obesity: Does it occur in African children in a rural community in South Africa?. Int. J. Epidemiol..

[15] Mashita R.J., Themane M.J., Monyeki K.D., Kemper H.C.G. (2011). Current smoking behaviour among rural South African children: Ellisras Longitudinal Study. BMC Pediatrics.

[16] Gunzerah I., Faden V., Zakhari S., Warren K. (2004). National institute on Alcohol abuse and alcoholism report on moderate drinking. Alcohol Clin. Exp. Res..

[17] Dhalla S., Kopec J.A. (2007). The CAGE questionnaire for alcohol misuse: A review of reliability and validity studies. Clin. Investig. Med..

[18] Ewing J.A. (1984). Detecting alcoholism: The CAGE questionnaire. JAM.

[19] Shelton N.J., Knott C.S. (2014). Association between alcohol calorie intake and overweight and obesity in England adults. Am. J. Public Health.

[20] Melchior M., Chastang J., Goldberg P., Fombonne E. (2008). High prevalence rates of tobacco, alcohol and drug use in adolescents and young adults in France: Results from the GAZEL Youth Study. Addict. Behav..

[21] Reddy S.P., Panday S., Swart D., Jinabali C.C., Amosun S.L., James S., Monyeki K.D., Stevens G., Morejele N., Kambaran N.S. (2003). Umthenthe Uhlaba Usamila—The South African Youth Risk Behaviour Survey 2002.

[22] Alamian A., Paradis G. (2012). Individual and social determinants of multiple chronic disease behaviour risk factors among youth. BMC Public Health.

[23] Department of Health, Medical Research Council (2007). OrcMacro: South. Africa Demographic and Health Survey 2003.

[24] Droomers M., Schrijvers C.T.M., Stronks K., van de Mheen D., Mackenbach J.P. (1999). Educational differences in excessive alcohol consumption: The role of psychosocial and material stressors. Prev. Med..

[25] Hemmingsson T., Lundberg I., Romelsjö A., Alfredsson L. (1997). Alcoholism in social classes and occupations in Sweden. Int. J. Epidemiol..

[26] Brinkley G.L. (1999). The causal relationship between socioeconomic factors and alcohol consumption: A granger-causality time series analysis, 1950–1993. J. Stud. Alcohol.

[27] Department of Health, Medical Research Council (1998). South. Africa Demographic and Health Survey 1998.

[28] Grant B.F., Dawson D.A. (1997). Age at onset of alcohol use and its association with DSM-IV alcohol abuse and dependence: Results from the National Longitudinal Alcohol Epidemiologic Survey. J. Subst. Abus..

[29] Dube S.R., Miller J.W., Brown D.W., Giles W.H., Felitti V.J., Dong M., Ada R.F. (2006). Adverse childhood experience and the association with ever using alcohol and imitating alcohol use during adolescence. J. Adolesc. Health.

[30] Valleman R., Templeton L. (2003). Alcohol Drugs and the family: Results from a long-Running Research Program within the UK. Eur. Adict. Res..

[31] Moser D.K., Kimble L.P., Alberts M.J., Alonzo A., Croft J.B., Dracup K., Evenson K.R., Go A.S., Hand M.M., Kothari R.U. (2006). Reducing delay in seeking treatment by patients with acute coronary syndrome and stroke. A scientific statement from the American Heart Association Council on Cardiovascular Nursing and Stroke Council. Circulation.

[32] Li Z., Bai Y., Guo X., Zheng L., Sun Y., Roselle A.M. (2016). Alcohol consumption and cardiovascular diseases in rural China. Int. J. Cardiol..

[33] Jardim T.V., Sousa A.L.L., Povoa T.R., Barroso W.S., Chinem B. (2014). Comparison of cardiovascular risk factors in different areas of health care over a 20 year period. Arq. Bras. Cardiol..

[34] Salaudeen A.G., Musa O.I., Babatunde O.A., Atoyebi O.A., Durowade K.A., Omokanye L.O. (2014). Knowledge and prevalence of risk factors for arterial hypertension and blood pressure pattern among bakers and traffic w ardens in Horin, Nigeria. Afr. Health Sci..

[35] Nedeltchev K., Fischer U., Arnold M., Kappeler L., Mattle H.P. (2007). Low awareness of transient ischaemic attacks and risk factors of stroke in a Swiss urban community. J. Neurol..

[36] Rimm E.B., Giovannucci E.L., Willett W.C., Colditz G.A., Ascherio A., Rosner B., Stampfer M.J. (1991). Prospective study of alcohol consumption and risk of coronary diseases in men. Lancet.

[37] Mikulík R., Bunt L., Hrdlicka D., Dusek L., Václavík D., Kryza J. (2008). Calling 911 in response to stroke: A nationwide study assessing definitive individual behaviour. Stroke.

[38] Liangpunsakul S., Crobb D.W., Qi R. (2010). Relationship between alcohol intake, body fat and physical activity—A population-based study. Ann. Epidemol..

[39] Boeckner L.S., McClelland J.W., Britten P., Chapman-Novakofski K., Mustian D., Clark C.D., Keim K.S. (1999). Evaluating diverse weight management programs with a standard evaluation questionnaire. JNE.

